# Unraveling the potential of phenomic selection within and among diverse breeding material of maize (*Zea mays* L.)

**DOI:** 10.1093/g3journal/jkab445

**Published:** 2022-01-17

**Authors:** Thea Mi Weiß, Xintian Zhu, Willmar L Leiser, Dongdong Li, Wenxin Liu, Wolfgang Schipprack, Albrecht E Melchinger, Volker Hahn, Tobias Würschum

**Affiliations:** 1 State Plant Breeding Institute, University of Hohenheim, Stuttgart 70593, Germany; 2 Institute of Plant Breeding, Seed Science and Population Genetics, University of Hohenheim, Stuttgart 70593, Germany; 3 Key Laboratory of Crop Heterosis and Utilization, Ministry of Education, Key Laboratory of Crop Genetic Improvement, Beijing Municipality, National Maize Improvement Center, College of Agronomy and Biotechnology, China Agricultural University, Beijing 100193, China

**Keywords:** phenomic selection, genomic selection, RR-BLUP, NIRS, predictive ability, landraces, maize breeding

## Abstract

Genomic selection is a well-investigated approach that facilitates and supports selection decisions for complex traits and has meanwhile become a standard tool in modern plant breeding. Phenomic selection has only recently been suggested and uses the same statistical procedures to predict the targeted traits but replaces marker data with near-infrared spectroscopy data. It may represent an attractive low-cost, high-throughput alternative but has not been sufficiently studied until now. Here, we used 400 genotypes of maize (*Zea mays* L.) comprising elite lines of the Flint and Dent heterotic pools as well as 6 Flint landraces, which were phenotyped in multienvironment trials for anthesis-silking-interval, early vigor, final plant height, grain dry matter content, grain yield, and phosphorus concentration in the maize kernels, to compare the predictive abilities of genomic as well as phenomic prediction under different scenarios. We found that both approaches generally achieved comparable predictive abilities within material groups. However, phenomic prediction was less affected by population structure and performed better than its genomic counterpart for predictions among diverse groups of breeding material. We therefore conclude that phenomic prediction is a promising tool for practical breeding, for instance when working with unknown and rather diverse germplasm. Moreover, it may make the highly monopolized sector of plant breeding more accessible also for low-tech institutions by combining well established, widely available, and cost-efficient spectral phenotyping with the statistical procedures elaborated for genomic prediction - while achieving similar or even better results than with marker data.

## Introduction

Plant breeding was revolutionized by the advent of genotypic information in the form of marker data. The seemingly obvious advantage was to introduce science to a subject formerly largely deemed as art (Jiang et al. 2020). This development has only occurred in the last decades with many approaches from quantitative trait loci (QTL) mapping (Würschum 2012) to genome editing developed since then (Benavente and Giménez 2021). Among these approaches is *genomic selection*, which was first implemented in animal breeding (Meuwissen et al. 2001), but has meanwhile also become a standard breeding tool for the prediction and subsequent selection of complex traits in plant breeding. Selection using genomic predictions was shown to lead to higher gains compared to pure phenotypic selection (Bernardo 2021b). There are different models that can be applied to perform genomic selection (Heffner et al. 2009) and many studies elaborating on, refining and comparing genomic selection approaches differing in their assumption with regard to the marker effect distribution are available (e.g. Resende et al. 2012; Thavamanikumar et al. 2015). Ridge regression best linear unbiased prediction (RR-BLUP), which assumes a homogeneous variance of all marker effects on the entire genome, has proven to be a robust method for predicting traits with many small-effect QTL (Heslot et al. 2012), as it generally results in high predictive abilities, expressed as the correlation between predicted and observed trait values. Besides QTL effects, the methods used for genomic prediction also exploit relatedness among individuals to achieve their predictive ability (Schopp et al. 2017; Bernardo 2021a). Special attention was also given to the role of the training set in genomic selection as its composition and relatedness to the prediction set are known to strongly impact prediction accuracies (e.g. Riedelsheimer et al. 2013; Schopp et al. 2015; Zhu et al. 2021). In general, genomic prediction is utilized to assist in a better use of available financial resources within the breeding process. It should be noted though, that generating marker data by genotyping is still cost-intensive and - if not outsourced - requires a certain laboratory infrastructure. There have been several advances and attempts in different crop species to include omics data other than genomics as predictors, but to date these are more difficult to obtain and more expensive than genotypic data (Riedelsheimer et al. 2012; Westhues et al. 2017; Schrag et al. 2018; Stich et al. 2020; Knoch et al. 2021).

Relatively new is the concept of *phenomic selection*, which was first proposed by Rincent et al. (2018) with the species poplar and wheat. The basic principle is to use near-infrared spectroscopy (NIRS) data instead of the marker data of each genotype for prediction. Hence, all wavelength information is used jointly. By this, the approach differs by definition from the traditional and ordinary NIRS application, which makes use of calibrations of only few wavelengths to predict specific traits (e.g. Melchinger et al. 1986). Several studies have successfully incorporated a set of wavelengths into the prediction models (Aguate et al. 2017; Hayes et al. 2017; Montesinos-López et al. 2017; Krause et al. 2019; Parmley et al. 2019; Galán et al. 2020); however, very few have investigated the innovative approach of phenomic selection as an alternative or complementary tool to facilitate selection decisions in breeding (Rincent et al. 2018; Lane et al. 2020). As a consequence, several questions with regard to the potential of applying phenomic selection in plant breeding still remain open. By providing answers to these questions, we might be able to broadly and routinely utilize this approach in breeding and thereby eliminate the need for genotyping (Lane et al. 2020). This might specifically empower less high-tech breeding companies and institutions, since an NIR spectrometer is rather easy to buy and maintain compared to a genotyping laboratory.

Hence, this study was motivated to contribute filling this gap, particularly regarding the performance of genomic and phenomic prediction within and among diverse groups of breeding material. To this end, we employed a set of 400 genotypes of maize - half elite material from two heterotic groups, half from 6 diverse landraces - to predict 6 traits relevant for maize breeding, i.e. anthesis-silking-interval, early vigor, final plant height, grain dry mattercontent, grain yield, and phosphorus concentration in the maize kernels. We particularly aimed to (1) estimate the predictive abilities of phenomic and genomic prediction within groups and among groups and to compare both approaches, (2) assess how population structure may influence predictive abilities of both approaches, (3) evaluate the impact of training set composition on the predictive abilities obtained by phenomic and genomic prediction, and (4) draw conclusions for practical plant breeding.

## Materials and methods

For a better overview, the different steps of data processing are visualized in Supplementary Fig. 1. Overall, 3 threads were defined, namely phenotypic, genotypic, and NIRS data processing.

### Phenotypic data

#### Field experiments

**Fig. 1.**
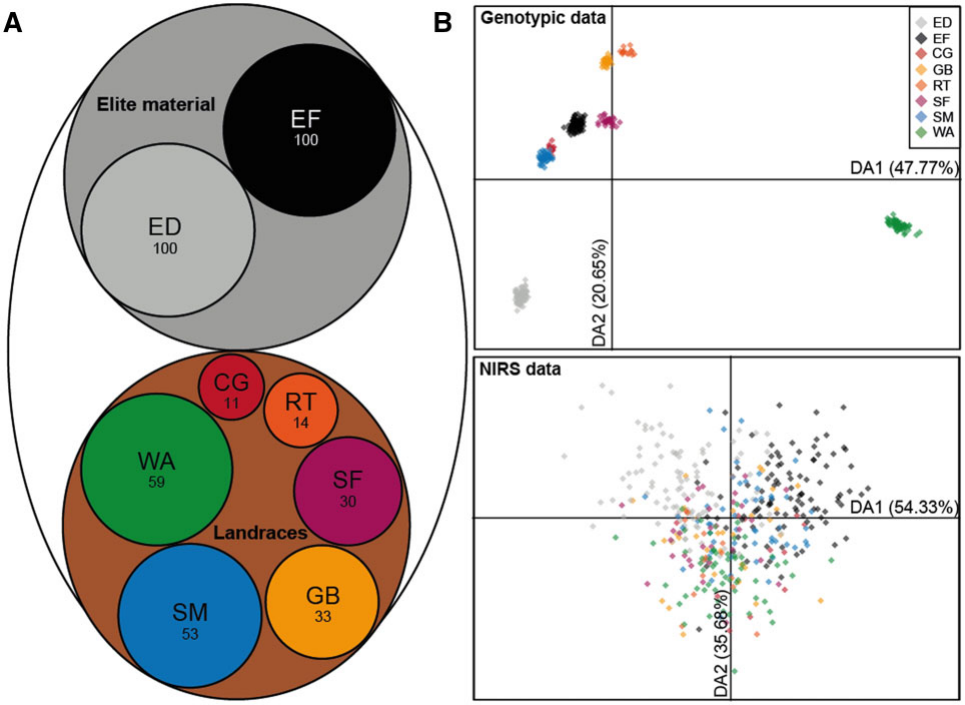
Population composition and discriminant analysis of principal components (DAPC). a) Elite material (*n* = 200) with ED and EF, landraces (*n* = 200) as a whole (LR) with CG, GB, RT, SM, SF, and WA. The size of each group is represented by the circle size and given as the number of individuals. b) DAPC of all 400 individuals from the 8 groups, performed with marker data (top) and NIRS data (bottom). The amount of variance explained by the first two discriminant analysis functions DA1 and DA2 is given in brackets.

In total, 400 genotypes were investigated in this study. These comprised 100 elite Dent (ED) lines, 100 elite Flint (EF) lines and 200 lines from 6 Flint landraces (LR) (Fig. 1a). The genotypes of the landraces group were immortalized as doubled-haploid lines and are comprised of the following 6 landraces: Campan-Galade (CG; *n* = 11) originating from France, Gelber Badischer (GB; *n* = 33) from Germany, Sankt Galler Rheintaler (RT; *n* = 14) from Switzerland, Satu Mare (SM; *n* = 53) from Romania, Strenzfelder (SF; *n* = 30) from Germany, and Walliser (WA; *n* = 59) from Switzerland. The plant material has been described in previous studies (Böhm et al. 2017; Würschum et al. 2021). Three different location-year-combinations served as environments for the phenotypic data used in this study, the experimental station for plant breeding in Hohenheim (HOH; 48°43′05.7″N, 9°11′20.8″E; 389 m above sea level) during the field seasons 2019 and 2020 and the experimental station Eckartsweier (EWE; 48°32′24.7″N, 7°51′15.1″E; 139 m above sea level) during the field season 2020. Average precipitation and mean temperatures over the last 5 years of the two locations amounted to 663.3 mm, 10.3°C and 683.3 mm, 11.5°C, respectively (Agrometeorology Baden-Württemberg 2021). The field trials were laid out as alpha lattices, designed with the software CycdesigN (VSN International 2018), and each genotype was replicated twice. Standard seed and field treatments were applied before and during the field season. The net plot size was 6 m^2^ and the sowing density was 8.66 plants/m^2^. The following 6 phenotypic traits were assessed in this study in all location-year-combinations corresponding to 2,400 plots in total: anthesis-silking interval (ASI; days between 50% pollen shedding and 50% visible silks of a plot), early vigor (EV; 1 = “very poor” to 9 = “very vigorous” score), final plant height (Final PH; for HOH measured as the mean of 3 single plants, for EWE as one estimated value over the whole plot, given in cm), grain dry matter content (in %), grain yield (GYield in t/ha), and P concentration in the maize kernels [Pconc; for HOH_2019 and EWE_2020; milled to 1 mm, measured by means of X-ray fluorescence (Bruker, Billerica, MA, USA) in ppm].

#### Best linear unbiased estimation

The raw data were subjected to the Bonferroni-Holm outlier detection (Bernal-Vasquez et al. 2016) using the R-package “multtest” (Pollard et al. 2005). The hereafter described mixed model was applied for the multiple environment analyses using the software ASRemL-R 3.0 (Butler et al. 2009):

(1) $$y_{\mathrm{ijkl}}=µ+g_{i}+e_{j}+{\left(ge\right)}_{ij}+r_{jk}+b_{\mathrm{jkl}}+\varepsilon_{\mathrm{ijkl}}$$

where the phenotypic trait value $y_{\mathrm{ijkl}}$ is explained by the overall mean µ, the factor genotype $g_{i}$, the location-year-combinations denoted here as environments $e_{j}$, the interaction of genotype and environment ${\left(ge\right)}_{ij}$, the design variables replication $r_{jk}$ and block effect $b_{\mathrm{jkl}}$ each nested within the environment, and the error term $\varepsilon_{\mathrm{ijkl}}$, for which a homogeneous variance was assumed.

By taking all factors as random into the model, broad-sense heritabilities (Cullis et al. 2006; Piepho and Möhring 2007) of the traits were calculated. For the case of calculating the group-specific variance components, we introduced dummy variables and derived broad-sense heritabilities for each group by the formula (Hallauer et al. 2010):

(2) $$H^{2}=\frac{\sigma_{g}^{2}}{\sigma_{g}^{2}+\frac{\sigma_{g\times e}^{2}}{n_{e}}+\frac{\sigma_{\varepsilon }^{2}}{n_{e}*n_{r}}}$$

where $\sigma_{g}^{2}$ denotes the genotypic variance in the group, $\sigma_{g\times e}^{2}$ denotes the group-specific genotype-by-environment interaction variance, $\sigma_{\varepsilon }^{2}$ denotes the variance of the error, and $n_{e}$ and $n_{r}$ denote the number of environments and replications, respectively. Using the factor genotype as fixed in the mixed model of Equation (1), we then calculated best linear unbiased estimates (BLUEs). These BLUEs were subsequently used as phenotypic trait values for all further analyses. The R-package “agricolae” was used to perform significance tests (α = 0.05) of the group means (de Mendiburu 2020). The phenotypic raw data as well as the hereof calculated BLUEs are provided in Supplementary Table 1.

### Genotypic data

All 400 genotypes were characterized with a 50K SNP array by Illumina (Ganal et al. 2011), resulting in total in 57,840 calls. Genotypic raw data are provided in Supplementary Table 2.

#### Quality control

As a first step, all marker data were filtered for their information content and 8,254 markers were found to only consist of missing values. The threshold of >50% for missing marker information and >5% for heterozygous state led to the exclusion of additional 844 markers and 637 markers, respectively. In addition, 2,222 monomorphic markers were detected of which 2,093 were not yet included in the missing and heterozygote filter steps and therefore also removed from the genotypic data, leaving 46,012 markers in the overall genotypic data. Next, we checked that no individual genotype fell under the criteria of having >20% missing marker data and/or >5% heterozygous markers. No filtering of genotypes due to these criteria was necessary. Finally, the genotypic data were split up according to the elite dent (*n* = 100), elite flint (*n* = 100), and landraces (*n* = 200) categorization. In each of these groups, markers containing only missing and/or monomorphic markers, and markers with a minor allele frequency (MAF) of <3% were once again removed from the subsets. Heterozygous marker information was in contrast to the complete marker data set not excluded but instead set to NA. All these filtering steps led to a total of 34,145 markers for the ED, 33,422 markers for the EF, and 38,284 markers for the LR group.

#### Imputation

The 3 groups were kept separately for imputation. Therefore, all 3 files were loaded into TASSEL (Bradbury et al. 2007) in HapMap format and transformed to the Variant Call Format. This format was then used for imputation by the software Beagle 5.2 (Browning et al. 2018). Standard settings for the imputation procedure were chosen, except for the effective population size *n_e_*, which was reduced to 1,000. After successful imputation, all 3 files were filtered and markers with MAF <5% were removed. The so obtained files were then merged, which resulted in a total of 17,845 imputed markers for the investigated 400 genotypes.

#### Genomic selection

We performed genomic selection with the R-package “rrBLUP” 4.6.1 with the basic model written as: y=WGu+ε (Endelman 2011), where the phenotypic value y is calculated by the product of the design matrix W, the genotype matrix G, and the vector u of marker effects with ε being the error term. Ridge regression keeps all markers in the model and shrinks their estimated effects by a constant factor (Whittaker et al. 2000). In general, we can distinguish between *predictions within* a group, where 5-fold cross-validation was performed and *predictions among* groups without cross-validation. Single landrace populations were only considered for ≥30 individuals. To perform 5-fold cross-validation, the dataset was for each run divided randomly into 80% training set and 20% prediction set. For the heterogeneous group “landraces”, consisting of 6 single populations, proportional sampling was performed per landrace and then the training set and the prediction set were combined accordingly. For within-group predictions, the number of cross-validation runs was 1,000, if not mentioned otherwise. The predictive ability was calculated as the Pearson correlation between predicted values and the BLUEs of each prediction set.

### NIRS data

NIRS data were obtained with a SpectraStar (Unity Scientific, Milford, MA, USA). The device covers the wavelength range between 1,250 and 2,400 nm with a stepsize of 1 nm (NIRS raw data are given in Supplementary Table 3 and are depicted in Supplementary Fig. 2a). In addition to seed samples from all 3 environments, NIR spectra of seedling biomass samples grown in EWE_2020 were recorded. Seed samples were obtained from ca. 375 g of the open-pollinated harvest sample used for the determination of grain dry matter content. All samples were ground to a final fineness of 1 mm filling a 200-ml tube (RETSCH GmbH, Haan, Germany). Of these subsamples, one NIR cup per plot was assembled and measured with 24 repetitions.

#### Quality control

First, the NIR spectra were cut at each side of the spectra by 18 nm to exclude undesired border effects, leaving 1,114 wavelengths to be further analyzed. Subsequently, Savitzky-Golay smoothing and first derivative of the NIR spectra were applied by means of the R-package “prospectr” 0.2.0 (Stevens and Ramirez-Lopez 2020). Afterwards, starting from wavelength 1,268 to wavelength 2,382, BLUEs were calculated of each single wavelength following the mixed model denoted in Equation (1). Heritabilities per wavelength were calculated (Supplementary Fig. 2b) and variance components per wavelength checked (Supplementary Fig. 2c).

#### Phenomic selection

All NIRS BLUEs were centered and scaled using the function “scale” in R before the so obtained values were subjected to further analyses. The standardization of the data is paramount for assuming a common variance of the regression coefficients (Piepho 2009). Exactly the same procedure as described for genomic prediction was carried out for phenomic prediction, except for using NIR data instead of marker data. Again, within group predictions were performed with 1,000 cross-validation runs. For predictions within and among groups, the two smallest populations with <30 individuals, namely CG and RT were not considered as a separate group. Hence, the following 7 groups were used for all genomic and phenomic predictions: elite Dents (ED), elite Flints (EF), landraces as a whole (LR) as well as the single landraces Gelber Badischer (GB), Satu Mare (SM), Strenzfelder (SF), and Walliser (WA).

If not specified otherwise, we used RStudio version 3.5.3 for all described data analyses (R Studio Team 2020) and the R packages “ggraph” (Lin Pedersen 2021), “ggpubr” (Kassambara 2020), “adegenet” (Jombart 2008) as well as basic R plot functions.

## Results

### Characterization of the material groups by phenotypic, genotypic, and NIRS data

The 400 genotypes that were investigated in this study can be differentiated into 8 groups: 100 elite lines of each of the two heterotic groups Flint and Dent, and 200 lines from 6 European Flint landraces (Fig. 1a). The discriminant analysis of principal components (DAPC) showed that the marker data reflected this underlying population structure, whereas no distinct clustering of the groups was apparent with NIR spectra (Fig. 1b).

**Fig. 2.**
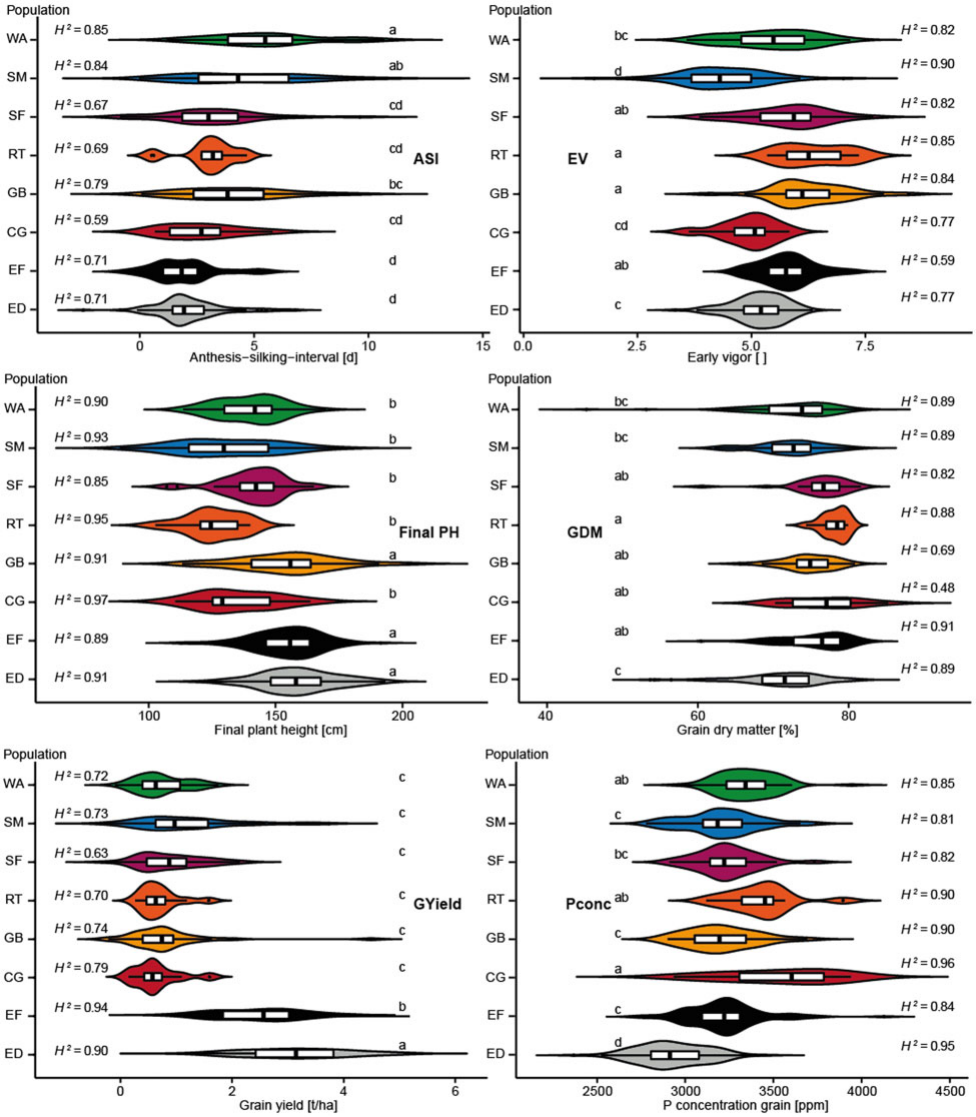
Phenotypic variation in the 8 groups. Groups are abbreviated as WA, SM, SF, RT, GB, CG, EF, and ED. Distribution of the phenotypic trait values for ASI (anthesis-silking-interval), EV (early vigor), Final PH (plant height at harvest), GDM (grain dry matter content), GYield (grain yield), and Pconc (phosphorus concentration in kernels) shown per group. The letter display indicates significant differences of the means; groups with the same letter are not significantly different from each other (α = 0.05). $H^{2}$ denotes the broad-sense heritability.

Furthermore, we assessed the phenotypic variation present in the plant material (Fig. 2 and Supplementary Table 4). This revealed that the single landraces exhibited significant differences between each other for certain traits and can therefore also phenotypically not be considered as one homogeneous group. As expected, the elite material showed on average significantly higher yields than the landraces. Overall, it could be observed that the higher the grain yield was, the lower were the P concentrations in the kernels. Notably, the elite Dent lines are generally later maturing under European field conditions than the elite Flint lines, which explains the grain dry matter content values and may also underlie their observed lower P concentrations in the maize kernels. Broad-sense heritabilities for the multienvironment analysis were overall high, ranging from 0.48 for grain dry matter content to 0.97 for final plant height, both in the landrace CG (Fig. 2 and Supplementary Table 4). Moreover, the genotypic variance components in each group illustrated the larger variation of landraces compared to elite material for the traits anthesis-silking interval, early vigor, and final plant height. For the trait grain yield, it was the other way around, with the broadest variation observed in elite material.

When correlating the BLUEs of the phenotypic data with those of the NIR spectra, we observed rather low correlations for most wavebands and different correlation patterns for the different traits (Supplementary Fig. 2d). Interestingly, opposed correlations for grain yield and P concentration with the NIR reflectance values were observed. Collectively, these results underpin that molecularly and phenotypically diverse breeding material was represented in this panel, which is therefore well suited to address the objectives of this study.

#### Predictions within groups

**Fig. 3.**
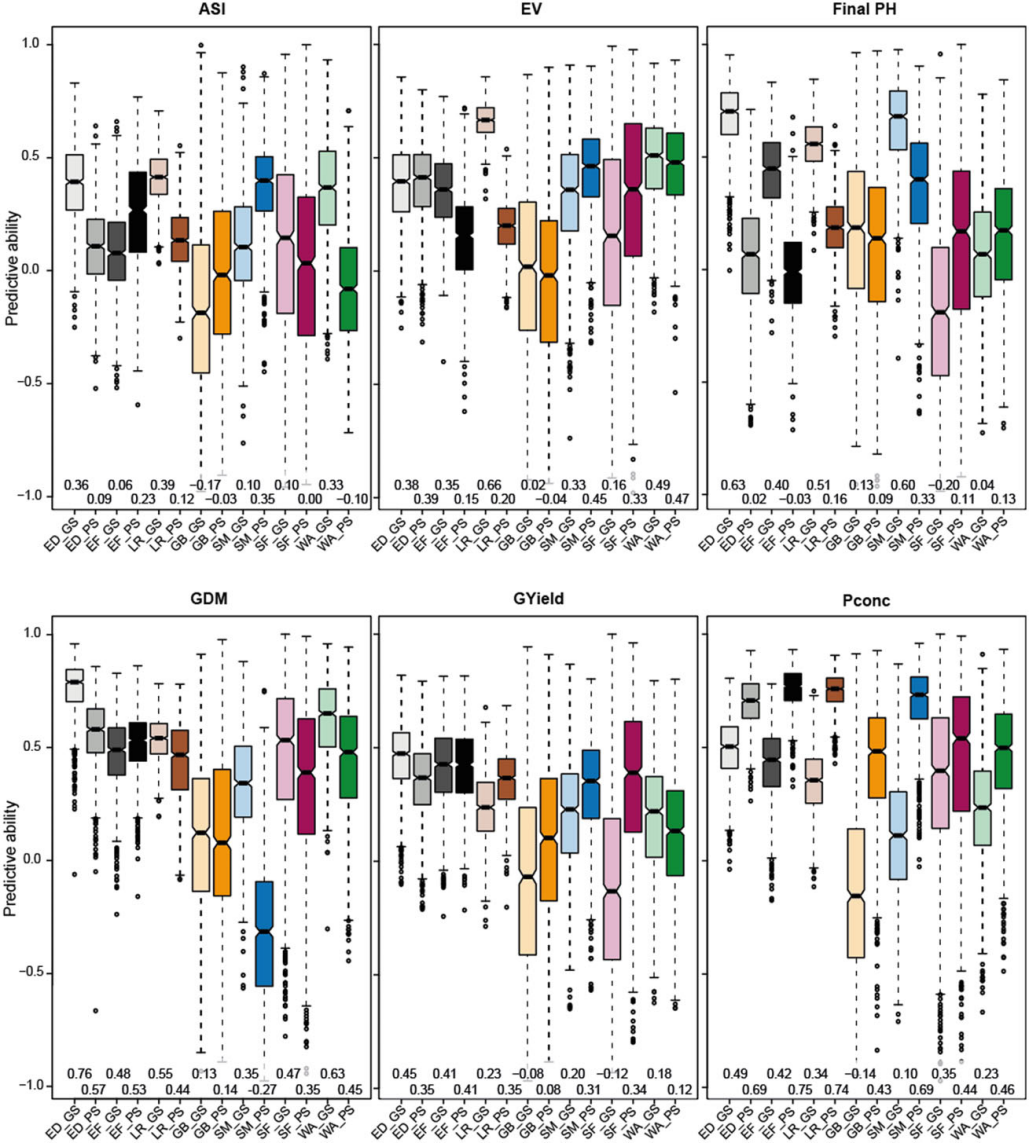
Multiple comparisons of the predictive abilities by within-group prediction. Cross-validated predictive abilities obtained by 1,000 runs are shown for each trait and group. Groups are abbreviated as ED, EF, all LR, GB, SM, SF, and WA. GS and PS abbreviate genomic and phenomic prediction, respectively. The mean of each scenario is given underneath the boxplots. Traits are denoted as ASI, EV, Final PH, GDM content, GYield, and Pconc.

We first performed genomic and phenomic prediction within groups (Fig. 3). This revealed no consistent pattern, as for most traits and groups the phenomic and genomic predictive ability was comparable, with sometimes one or the other being better, but often only slightly. Genomic prediction achieved generally better results for the traits final plant height and grain dry matter content. Conversely, grain yield was overall better predicted by phenomic prediction and for the trait P concentration phenomic prediction outperformed genomic prediction substantially for all groups. The predictive ability values obtained by cross-validation, confirmed for genomic and phenomic prediction alike that smaller groups (the landraces GB, SM, SF, and WA), and therefore smaller training and prediction sets, resulted in lower mean predictive abilities with a generally larger variation.

**Fig. 4.**
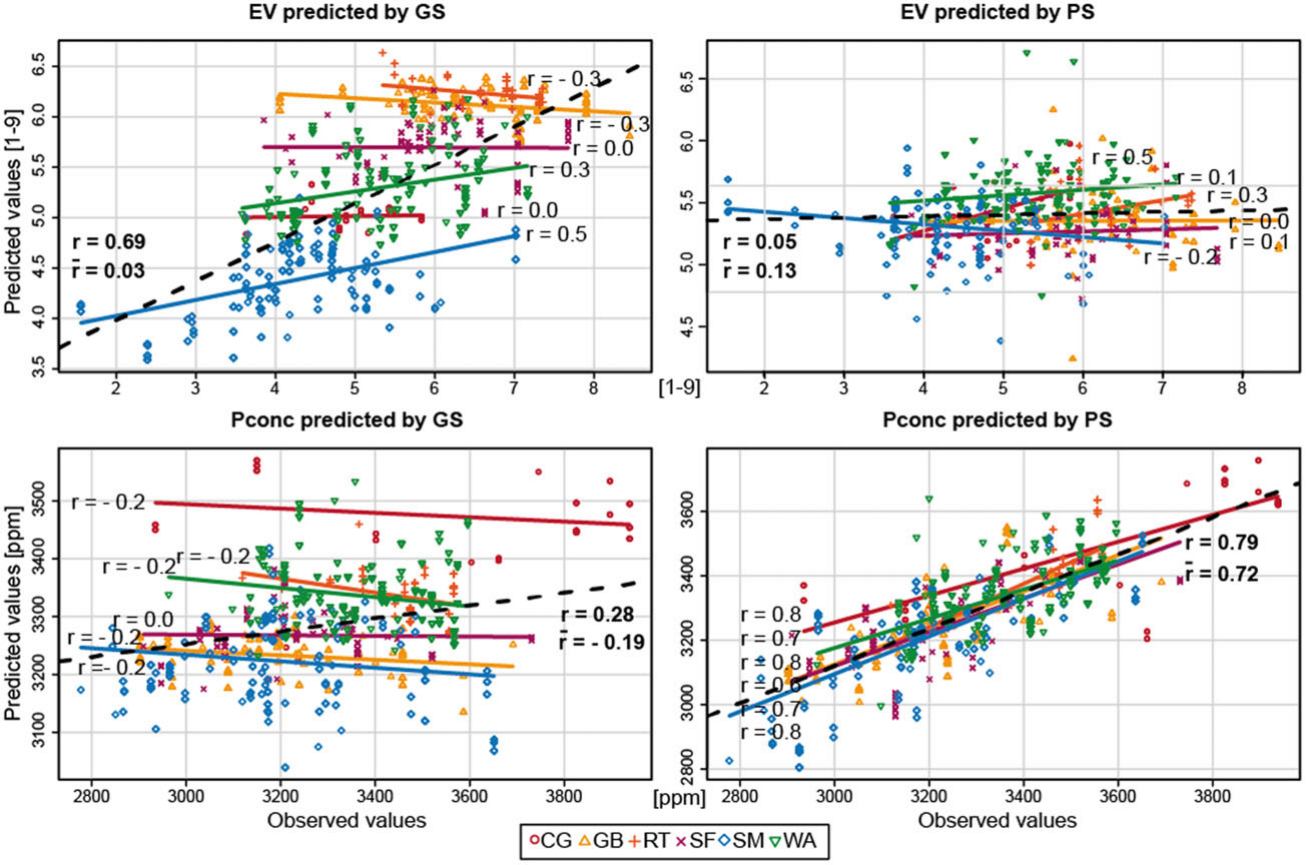
Predictive ability in the heterogenous group of landraces. Comparison of genomic (GS) and phenomic (PS) prediction for the traits EV and Pconc. For EV, the genomic predictive ability was much higher, whereas for Pconc the phenomic approach resulted in a much higher predictive ability than genomic prediction. The Pearson correlation coefficients within each group are indicated as r. In bold, the Pearson correlation coefficient across all genotypes is given and $\overset{-}{r}$ denotes the mean of all correlation coefficients of the single landraces. The dots represent the observed and the predicted trait values from 10 cross-validation runs.

We then focused on the cases where we observed the most prominent discrepancies between the genomic and the phenomic prediction approach in the 3 major groups. Large differences between both approaches were observed for predictions in the group of landraces. For early vigor, genomic prediction outperformed phenomic prediction by 0.46. On the other hand, P concentration was characterized by a 0.40 higher phenomic predictive ability compared to genomic prediction. In order to illustrate the cause for these differences, we plotted for 10 cross-validation runs the predicted and observed values of the traits early vigor and P concentration for each individual genotype of the 6 landraces (Fig. 4). For early vigor, the overall correlation coefficient of the genomic approach was relatively high with *r* = 0.69. However, the mean trait performance was quite different among the landraces and the correlations assessed within them only averaged to $\overset{-}{r}$ = 0.03. This low predictive ability within each landraces was reflected by phenomic prediction, for which the overall correlation coefficient r was indeed low with 0.05, hence only slightly deviating from the average $\overset{-}{r}$ across the landraces that was 0.13 and thus even higher compared to the genomic approach ($\overset{-}{r}$ = 0.03). In the case of P concentration for which phenomic prediction yielded a much higher predictive ability than genomic prediction, the genomic approach resulted in low within-landrace correlation coefficients ($\overset{-}{r}$ = −0.19), but also a low overall correlation (*r* = 0.28), as the phenotypic differences among the landraces were minor. By contrast, phenomic prediction showed a high overall correlation coefficient of *r* = 0.79, which was again mirrored by the correlation coefficients observed in each single landrace ($\overset{-}{r}$ = 0.72).

#### Predictions among different groups

**Fig. 5.**
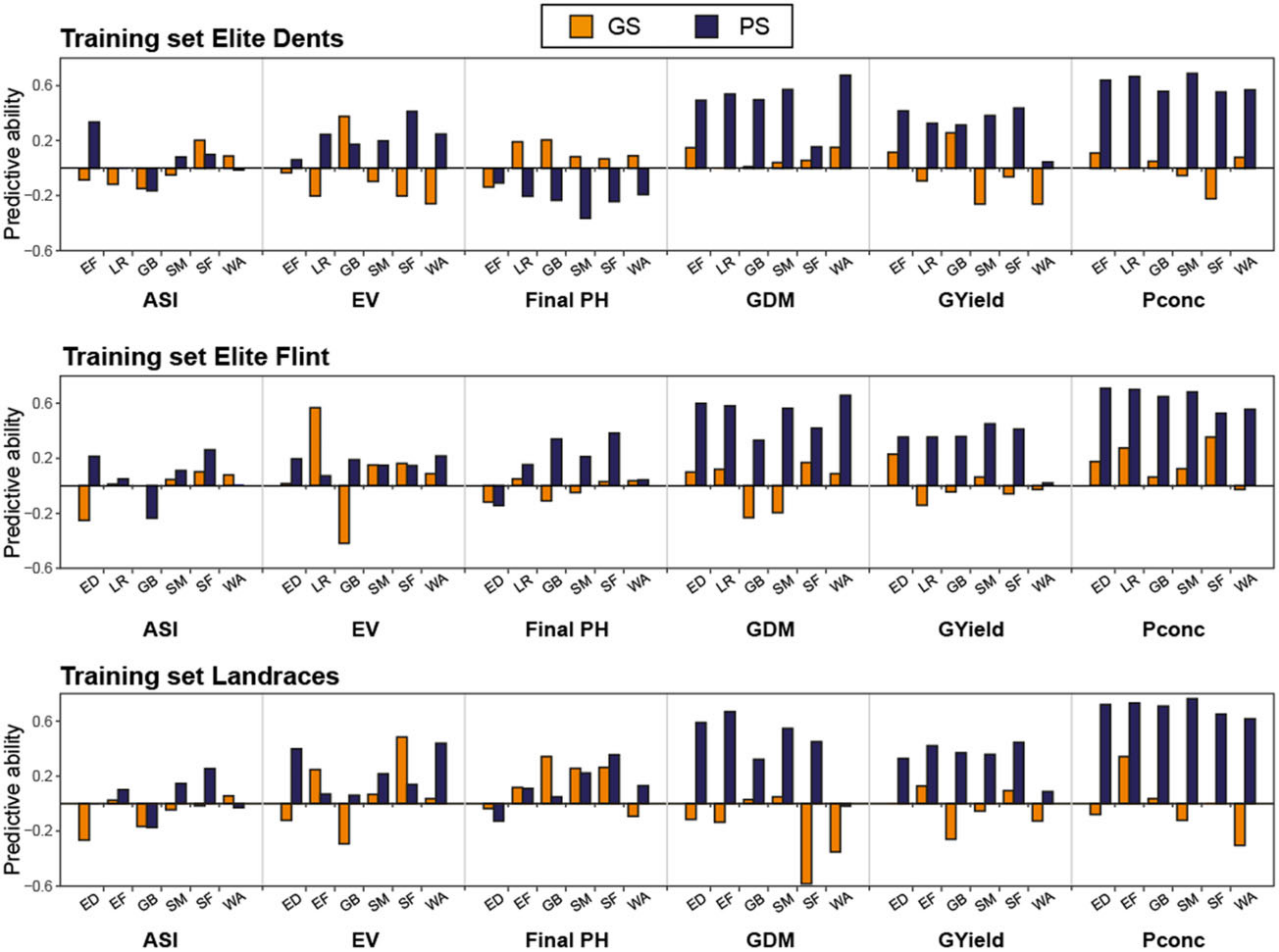
Predictive ability of among-group predictions. Results are shown for the ED, EF or the landraces being used as training set for genomic (GS) or phenomic (PS) prediction to predict each of the other groups. Groups are abbreviated as ED, EF, all LR, GB, SM, SF, and WA. Traits are denoted as ASI, EV, Final PH, GDM content, GYield, and Pconc.

We next assessed predictions among groups, i.e. using one group as training set and another one as prediction set (Fig. 5 and Supplementary Figs. 3 and 4). In general, the phenomic predictive abilities surpassed the genomic ones when one of the 3 major groups was used as training set. An exception was the prediction of final plant height when using elite Dents as training set, as for this scenario the predictive ability was negative for prediction in all other groups, whereas positive predictive abilities were achieved for the genomic approach except for the elite Flints. However, especially for the traits grain dry matter content, grain yield, and P concentration, phenomic prediction yielded substantially higher predictive abilities compared to genomic prediction. The same trends as observed for the 3 major groups, though potentially slightly less pronounced, were observed when the two largest landraces, SM or WA, were used as training set (Supplementary Fig. 3). Phenomic prediction yielded overall more stable results, which can also be well seen in the visualization of the predictive abilities separated by group (Supplementary Fig. 4). Here, we can also conveniently compare reciprocal predictions, which substantiated the much lower robustness of genomic compared to phenomic among-group predictions. The phenomic prediction results, by contrast, yielded similar patterns no matter in which direction the prediction took place.

Phenomic predictions were also performed with NIR spectra of maize seedling biomass for the environment EWE_2020. Predictions based on biomass generally achieved lower predictive abilities than seed-based ones. This held true for the comparisons of the seed NIRS BLUEs across all 3 environments (results not shown) as well as for the seed NIRS data of EWE_2020 alone (Supplementary Table 5).

In summary, phenomic prediction resulted in much higher predictive abilities than genomic prediction for the prediction among groups.

#### Evaluation of composite training sets

**Fig. 6.**
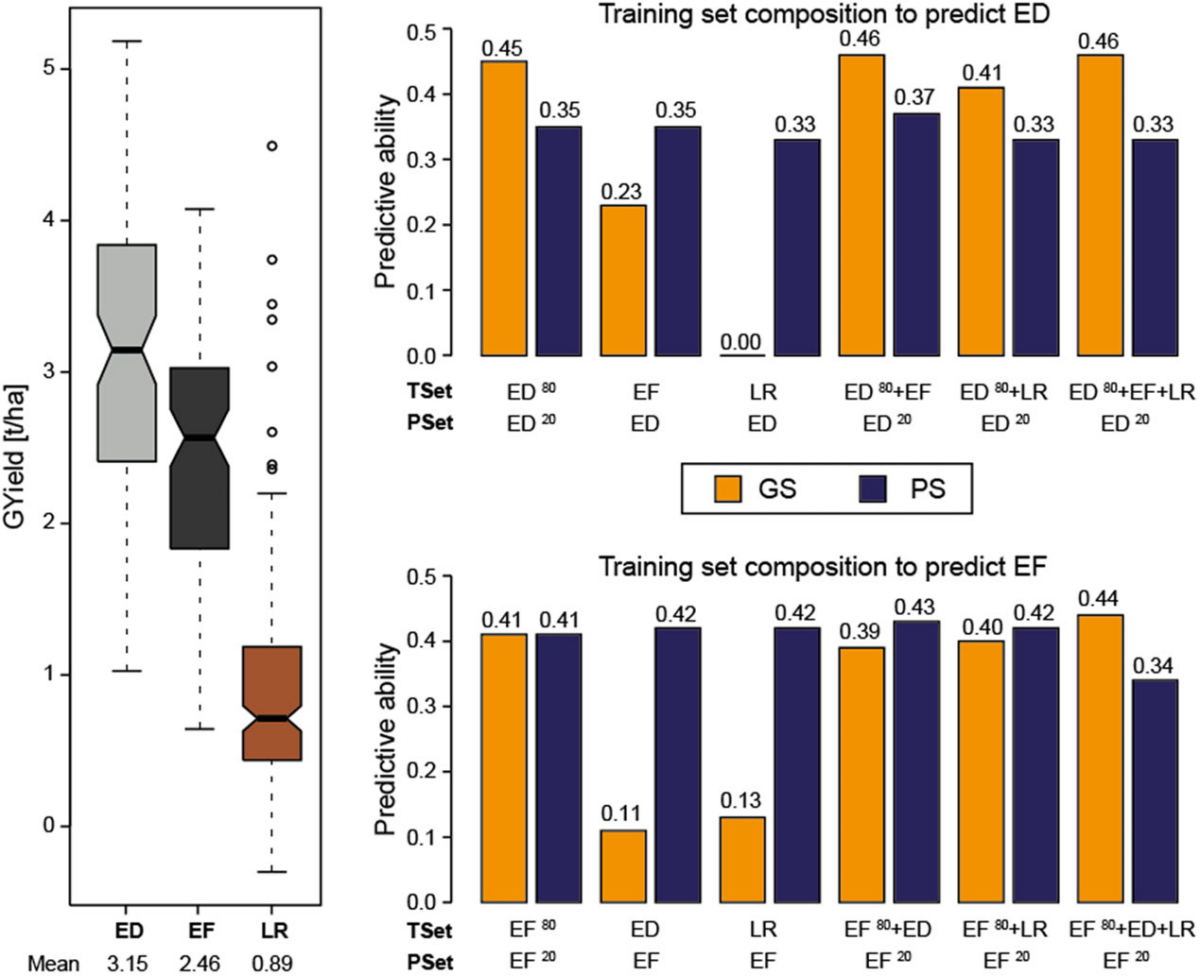
Evaluation of composite training sets. For the 3 major groups, ED, EF, and landraces, different training set compositions were tested for grain yield for genomic (GS) and phenomic (PS) prediction. The boxplot on the left shows the differing level of phenotypic performance for the 3 groups. The bar plots on the right depict the predictive ability of the different scenarios for either the ED (top) or the EF (bottom). The superscript numbers 80 and 20 reflect the proportion of individuals in the training set (TSet) and the prediction set (PSet). Predictions within the respective elite groups were obtained with 1,000 5-fold cross-validation runs; for the composite training sets, 100 cross-validation runs were used.

The trait grain yield was chosen to investigate the potential of combining groups into composite training sets, which were then larger but also composed of material from different groups with different trait performance (Fig. 6). To predict elite Dents and elite Flints, the following scenarios were compared with each other: (1) 5-fold cross-validated prediction within the respective elite group, (2) across prediction from the other elite group as well as from the landraces group as a whole, and (3) combinations of 80% of the lines from the elite group to be predicted with one or both of the other major groups to predict the 20% remaining lines of the respective elite group. Phenomic prediction resulted in highly similar predictive abilities for all 6 scenarios for both the elite Flint and elite Dent lines. As shown before, genomic prediction was on a comparable level for the cross-validated within-group prediction, but performed poorly for the among-group prediction. Interestingly, genomic prediction then resulted in similar or even slightly higher predictive abilities compared to phenomic prediction for the 3 composite training sets. The predictive abilities achieved with these composite training sets were similar to that obtained by the within-group prediction.

## Discussion

### Comparison of genomic and phenomic prediction

First, it has to be stated that a high quality of phenotypic data is and will remain the basis for all breeding activities (Bernardo 2021a). This was given in this dataset, which showed a large genotypic variation and high to very high trait heritabilities in the multienvironment analysis (Supplementary Table 4).

Looking at the obtained cross-validated predictive abilities within groups, genomic and phenomic prediction generally yielded comparable results (Fig. 3). This performance was, however, also dependent on the trait (Knoch et al. 2021) and the genetic group. It should be mentioned here, that no general conclusion should be drawn solely from the results of the single landraces with their rather small population sizes. Final plant height, for example, was much better predicted by marker than by NIRS data, but mainly in the elite material. This material is somewhat taller than the landrace lines, but otherwise there is no apparent difference. For grain dry matter content, the difference in predictive ability was most pronounced for the elite Dents and the landrace SM from Romania, which are the two latest maturing groups as can be seen by their lowest means for this trait (SM = 72.81%, ED = 70.88%). All seed samples were completely dried before NIR spectra were measured and one might assume that drying maize kernels containing more water at the beginning might change their properties in a way that altered the NIRS assessment. However, for other traits like early vigor or grain yield, phenomic prediction was as good or even better than genomic prediction for these two groups. For grain yield, we found that both approaches performed similarly, which is promising as grain yield is a central trait in every breeding program. In line with this, Lane et al. (2020) reported prediction abilities above 0.7 for grain yield obtained by phenomic prediction of whole-kernel maize samples. For the trait P concentration, we observed higher phenomic predictive abilities compared to the genomic approach for all groups. Notably, P concentration is also an endophenotype of the seeds, which may have contributed to this performance.

Taken together, the reasons for the discrepancies observed between the two approaches for some traits and groups are not clear and require further research. Nevertheless, our results confirmed the potential of phenomic prediction for NIRS-assisted selection in breeding, as the phenomic predictive abilities were generally competitive with those from genomic prediction. NIRS data can already be obtained from early- or mid-generation selection candidates before any yield trials in multiple environments have been performed. Phenomic prediction can thus be used to predict more resource-intensive traits such as grain yield and thereby assist the identification of the most promising candidates to be advanced to the next generation.

#### Population structure can lead to overestimation of the genomic predictive ability

The greatest differences between the genomic and phenomic predictive abilities were often observed for the panel of landraces, where for the 3 traits anthesis-silking interval, early vigor, and final plant height genomic prediction appeared to perform much better. We exemplarily used early vigor to further investigate this different performance. Analyzing the correlations between the observed and the predicted trait values per landrace revealed that the high predictive ability of early vigor by genomic prediction was an artifact (Fig. 4). While the overall correlation coefficient was high with 0.69, each single landrace showed only weak correlations that averaged 0.03. The reason for this is the confounding of population structure and trait performance, here in the form of the different landraces and their mean performance, a phenomenon which has been described in previous studies (Windhausen et al. 2012). All 3 traits showed clear differences in their means among the landraces (Fig. 2). In addition, the marker data can clearly separate landraces as shown by the discriminant analysis of principal components (Fig. 1B). In contrast to the marker data, the NIRS data do not distinguish the LR as groups in the DAPC as clearly as the marker data and are therefore less prone to this kind of artifact. For the NIRS data, the low overall correlation coefficient for early vigor of 0.05 much more accurately reflected the predictive ability in the single landraces.

For the reverse case of phenomic prediction outperforming genomic prediction in the landraces for the trait P concentration, by contrast, the high overall correlation coefficient of the former correctly portrays the high predictive ability in each of the landraces. In summary, this confirms previous results concluding that genomic selection is sensitive to population structure (Thorwarth et al. 2017). As a consequence, seemingly high genomic predictive abilities achieved with panels showing population structure should be interpreted with caution and always in combination with the trait performance of the populations.

#### Phenomic prediction works well among different breeding material

A major advantage of phenomic prediction became apparent when predicting from one group to another (Fig. 5). Genomic prediction has been described to strongly depend on the relatedness between training and prediction set (Albrecht et al. 2011; Riedelsheimer et al. 2013; Li et al. 2021; Zhu et al. 2021). Our results corroborate these findings, as the prediction among groups resulted in only low predictive abilities, even for the predictions among the more closely related Flint material. The phenomic predictive abilities, on the contrary, were much higher, especially for the traits grain dry matter content, grain yield, and P concentration. For these 3 traits, the among-group predictive abilities were often as high as the cross-validated within-group ones.

Collectively, these findings illustrate that phenomic prediction is very promising for rather diverse breeding material with more or less unrelated groups, whereas genomic prediction has been shown to work best if the material in the training and prediction set are from the same group (Schopp et al. 2015).

#### Composition of the training set with diverse breeding material

These findings rose the question on how different compositions of the training set would affect the prediction of grain yield of the elite lines. Notably, grain yield differed substantially between the elite material and the landraces. As phenomic prediction appeared to be tolerant to unrelated material being used as training set, we hypothesized that increasing the training set with lines from other material groups than the one to be predicted would improve or at least not hamper the predictive ability of phenomic prediction. While the other elite group and the landraces both yielded predictive abilities similar to the cross-validated within-group phenomic predictions, adding them to the training set did not enhance the predictive ability. For the prediction of both elite groups, the phenomic predictive abilities were more or less unchanged for all tested scenarios of training set composition. It might be that even though the predictive abilities of all 3 groups are highly similar, the effect estimates are different and combining them does not yield an advantage or that even with the smallest training set size of the 80 lines (80%) sampled from the same group, the predictive ability already reached a plateau stage. Our results of phenomic predictive abilities between 0.33 and 0.43 with combined training sets are consistent with a former study, which reported a phenomic prediction ability based on maize kernel NIRS of on average 0.28 for grain yield in elite material, when a diversity set and 10% of each group to be predicted were used as training set (Lane et al. 2020).

While the genomic prediction using one of the other two major groups virtually failed, adding them to the training set resulted in similar or even slightly higher predictive abilities as the cross-validated within-group predictive ability. This is in line with previous findings that showed that adding less related lines to a training set did not reduce the predictive ability (e.g. Brauner et al. 2020; Li et al. 2021; Zhu et al. 2021) and increasing the training set size generally results in higher predictive abilities of genomic selection (e.g. Zhao et al. 2012; Thorwarth et al. 2017; Li et al. 2021; Zhu et al. 2021).

Nevertheless, if all scenarios are considered, phenomic prediction showed a higher robustness of the predictive abilities for different compositions of the training set and thus relatedness between training and prediction set. This result is also worth mentioning as it suggests that the general assumption that predictive breeding strongly relies on estimating the genetic relatedness among individuals (Bernardo 2021a) may not hold true for the approach of phenomic prediction.

#### Possible effects of the NIRS sample material on the trait prediction

Spectral data can not only be obtained from seeds but also from other plant material, thereby potentially allowing selection at different stages of a breeding program. We derived NIR spectra from maize kernels as well as for one environment from seedling biomass samples, both ground to 1 mm. In our study, the results obtained with the seed sample NIRS data were generally better (Supplementary Table 5). This is in line with previous findings that in addition showed higher genotypic variances for grain in comparison to leaf samples, which may underlie the higher predictive abilities of the former (Rincent et al. 2018). Interestingly, when looking at the single traits separately, we observed higher predictive abilities based on NIRS of seedling biomass for early vigor and final plant height, whereas anthesis-silking-interval, grain dry matter content, grain yield and P concentration were clearly better predicted by seed samples. The picture this presents is that the closer the sampled tissue is in relation to the trait of interest, the better the prediction works based on this tissue. Our data set is clearly too small to substantiate this conclusion, but this warrants further research. We also correlated the NIRS BLUEs of the seedling biomass with the trait BLUEs for each genotype, but could not discern a pattern between these correlations and the goodness of the trait prediction (data not shown). It should be noted here that grain yield is often the most important trait for breeders and therefore seed-based NIR spectra appear more promising for application in breeding programs.

#### Application of phenomic prediction in practical breeding

Rincent et al. (2018) showed in simulations that for different scenarios regarding the costs and reliabilities of phenomic and genomic prediction, the expected selection gain was in most cases higher for phenomic compared to genomic selection. The advantages of performing predictions based on NIRS data compared to genotypic data are the low requirements for infrastructure such as specialized laboratories and the strongly reduced costs, both coupled with the benefit of an increased speed and efficiency of selection in the breeding program. The first point challenges the assumption that became prevalent in the last years, namely that the most cost-efficient tool for trait prediction is found in genetic marker data (Bernardo 2021b; Knoch et al. 2021). Supposed that seeds represent the most suitable material to obtain NIR spectra, we are much faster to capture all data with a state-of-the-art spectrometer compared to the DNA extraction and subsequent genotyping. If we have to take decisions that are time-critical, as for example, frequently encountered in winter cereals between harvest and sowing or in maize in between shuttle breeding seasons, phenomic prediction results can be obtained quicker than genomic prediction results. Moreover, the phenomic selection approach appears particularly attractive for comparatively low-tech institutions because less investments and resources are needed for NIRS measurements compared to genotyping.

A main finding of this study is that phenomic prediction also reliably works for designs of training sets that show a population structure as well as for the case that the training set and the prediction set include less related material. In breeding programs that stay within their established material, as for example in maize within the existing heterotic groups, this is of no relevance. So, when could this become relevant in practical breeding? For instance, if we were to start a breeding program in a new environment by combining material from different origin. After an initial field evaluation and yield trials with the labor- and/or resource-intensive traits being assessed only on a subset of lines, phenomic prediction based on the NIR spectra of the harvested material of all lines could support the identification of further candidates to be more intensively tested. Likewise, broadening the genetic basis of a breeding program by introgression of less related material from different groups, as exemplified here with the landraces, will require testing this material but may also profit from the additional prediction of the expected performance. Furthermore, crops with yet undefined heterotic pools or the presence of subpopulations such as wheat (Boeven et al. 2016) or sorghum (da Silva et al. 2021) could benefit from the independence of the performance of phenomic prediction with regard to the training set composition and its possibly underlying population structure. In addition, breeding programs are driven by the different selection cycles, which by nature imply a decreasing relatedness of the individuals from one cycle to the next (Schopp et al. 2015; Auinger et al. 2021). Further research is required to investigate whether phenomic prediction can provide higher predictive abilities than genomic prediction when using the current cycle as training set for the prediction of the individuals of the next cycles. Eventually, the decision of whether to use the genomic or the phenomic approach will be made in very practical terms, depending on the available resources and infrastructure, as well as the characteristics of the particular breeding program.

## Conclusion

While a large number of studies is available on different aspects of genomic prediction, the use of NIRS or other spectral data for phenomic prediction is still in its beginnings. We therefore compared both approaches under different scenarios for a set of 6 traits relevant in maize breeding. Apart from being cost-efficient and amenable to high-throughput, the potential of phenomic selection lies in its reliable predictions also under structured training sets as well as for predictions into unrelated material. In the end, however, it is not about a competition of the two approaches, but rather to expand the breeder’s toolbox and ideally one can choose from different approaches the one that is best suited for a given situation. Thus, resources can be allocated in the best possible way in order to maximize selection gain. Just as seen for genomic prediction over the past years, further research is required to better understand and refine the approach of phenomic prediction toward a broader application in plant breeding. Specifically, NIRS sample material and the environmental effect on the spectra should be investigated in the future. Nevertheless, our results demonstrate the value of phenomic prediction as a low-cost and efficient tool to support selection of complex traits in plant breeding.

## Data Availability

The authors affirm that all data necessary for confirming the conclusions of this article are represented fully within the article and its tables and figures. Supplemental material is included at figshare: https://doi.org/10.25387/g3.16692409.
